# Oligopeptides as Biomarkers of Cyanobacterial Subpopulations. Toward an Understanding of Their Biological Role

**DOI:** 10.3390/toxins6061929

**Published:** 2014-06-23

**Authors:** Ramsy Agha, Antonio Quesada

**Affiliations:** Departamento de Biología, Universidad Autónoma de Madrid, C/Darwin, 2, Madrid 28049, Spain; E-Mail: antonio.quesada@uam.es

**Keywords:** secondary metabolites, non-ribosomal, chemotypes, MALDI, NRPS, polymorphism, diversification

## Abstract

Cyanobacterial oligopeptides comprise a wide range of bioactive and/or toxic compounds. While current research is strongly focused on exploring new oligopeptide variants and their bioactive properties, the biological role of these compounds remains elusive. Oligopeptides production abilities show a remarkably patchy distribution among conspecific strains. This observation has prompted alternative approaches to unveil their adaptive value, based on the use of cellular oligopeptide compositions as biomarkers of intraspecific subpopulations or chemotypes in freshwater cyanobacteria. Studies addressing the diversity, distribution, and dynamics of chemotypes in natural systems have provided important insights into the structure and ecology of cyanobacterial populations and the adaptive value of oligopeptides. This review presents an overview of the fundamentals of this emerging approach and its most relevant findings, and discusses our current understanding of the role of oligopeptides in the ecology of cyanobacteria.

## 1. Introduction

Cyanobacteria are among the oldest and most successful forms of life still present on Earth. Ancient cyanobacteria are believed to have been instrumental in the transition of the atmosphere to oxic conditions. Today, these phototrophic prokaryotes are present in almost every habitat on Earth, including terrestrial, brackish, marine, and freshwater environments. They can be found in extreme environments ranging from arid desert areas and polar regions, to the inside of rocks (endolithic), or as symbionts with other organisms. The remarkable adaptability of cyanobacteria is attributable, in part, to the production of a wide repertoire of secondary metabolites with diverse bioactive activities, which likely contributed to the colonization of such a range of ecological niches. 

The term secondary metabolism is generally used to refer to metabolic pathways that are not directly involved in the growth, development or reproduction of the organism. In the case of cyanobacteria, secondary metabolites present a vast chemical diversity and are widespread across cyanobacterial taxonomic units. A growing number of these compounds has been isolated and characterized from cultured strains and field samples. Their discovery has been driven by the growing interest toward their potential pharmacological applications or, alternatively, their toxic effects on human and animal health. Resulting from this dual interest, we perceive in the literature a somewhat artificial separation of these metabolites into cyanotoxins and other bioactive compounds. This dichotomy has largely influenced the perspective by which the study of cyanobacterial secondary metabolites has been approached in the last decades. Exceptions notwithstanding, much of the research effort has targeted these compounds toward their potential pharmacological uses, e.g., as antibacterial, antiviral, anticoagulant or anticancer compounds [1,2,3,4,5], whereas their adaptive value for the producing organism has remained comparatively less studied. In the case of cyanotoxins, putative ecological roles have been explored with greater interest, fueled by water management and public health concerns resulting from the increasing occurrence of harmful cyanobacterial blooms in aquatic ecosystems worldwide [6,7]. However, even in these studies a clear distinction between cyanotoxins and other bioactive compounds is often made, hampering more integrative interpretations of the existing link between the secondary metabolism and the ecology of cyanobacteria. In fact, whereas research on cyanotoxins has produced a number of hypotheses on their adaptive value, at present, none of them is free of controversy (e.g., [8,9,10]). 

The discovery of intraspecific polymorphisms in picoplanktonic cyanobacteria has stimulated the perception that addressing the species as the lowest taxonomic level might be insufficient to understand the ecological plasticity of cyanobacteria. *Synechococcus* and *Prochlorococcus* populations were shown to subdivide into distinct ecotypes with different niche preferences [11,12,13,14]. Population subdivision allows these genera to rapidly adapt to a range of environmental conditions, which is regarded as one the major reasons behind their widespread distribution and ecological success [15].

In other cyanobacteria, the existence of intraspecific polymorphisms with regard to the synthesis of secondary metabolites is not a new notion. However, chemical polymorphisms have been mostly addressed in relation to the co-existence of toxigenic (*i.e*., toxin producing) and non-toxigenic strains exclusively [16,17,18], neglecting other “non-toxic” metabolites, and thereby sticking to the traditional separation of cyanotoxins and other bioactive compounds. In the last decade, however, more integrative perspectives have overcome this boundary by addressing the occurrence of both cyanotoxins (e.g., microcystins) and other bioactive peptides indistinctively and using them as subpopulation biomarkers, based on the overarching hypothesis that the secondary metabolism in cyanobacteria is an important feature closely coupled to their ecology [19,20].

Research focusing on the study of cyanobacterial chemotypes and their differential behavior under natural conditions has yielded unique insights into the composition and dynamics of natural populations. Furthermore, chemotyping approaches have led to the formulation of new hypotheses regarding the role of cyanobacterial oligopeptides, which will most likely shape future research efforts exploring their involvement in the ecology of cyanobacteria. Our intent here is not to recreate some excellent reviews comprehensively addressing the biosynthesis, chemical diversity, or bioactive properties of these compounds (e.g., [21,22,23,24,25]). Rather, we seek to review the fundamentals of oligopeptide-based chemotyping approaches in cyanobacteria and discuss the major findings stemming from their use. We also aim to highlight how the search for ecologically functional units below the species level is contributing to increase our understanding of the structure and dynamics of cyanobacterial populations and the putative adaptive value of their strongly diversified secondary metabolism.

## 2. Cyanobacterial Oligopeptides

**Figure 1:**
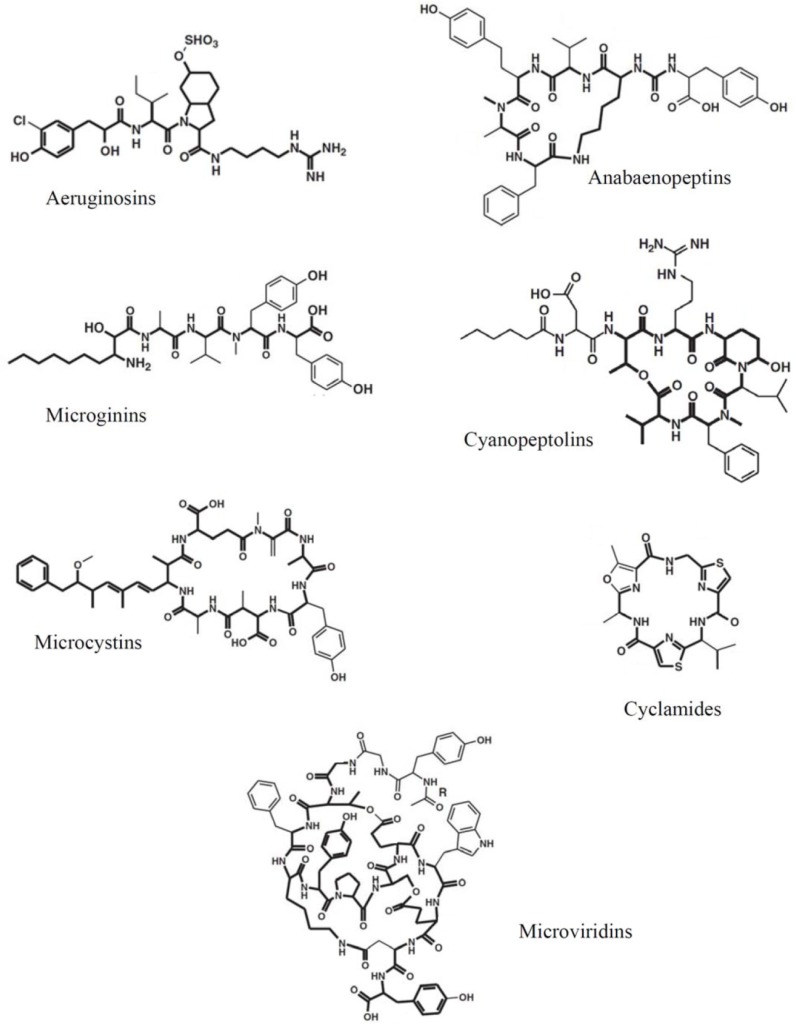
Examples of chemical structures of the seven major oligopeptide classes after Welker and Von Döhren [26]. Bold lines stand for conserved substructures within oligopeptide class. Thin lines stand for variable parts of the molecules that give rise to the existence of multiple chemical congeners within oligopeptide classes.

A major part of the array of secondary metabolites produced by cyanobacteria is represented by oligopeptides, a highly diverse group of low molecular weight peptides containing a range of both proteinogenic and non-proteinogenic amino acids. Oligopeptides display a surprisingly high level of structural variability, although many compounds share in common conserved substructures. These structural similarities led to the current, most widely accepted classification of oligopeptides, proposed by Welker and von Döhren [26], which establishes seven major peptide classes: aeruginosins [27], cyanopeptolins [28], anabaenopeptins [29], microginins [30], microviridins [31], cyclamides [32], and the well-studied microcystins [33], known for raising public health concerns as a result of their toxic effects on mammals (Figure 1). 

Most oligopeptides are synthesized following non-ribosomal pathways (e.g., [34,35,36]), although ribosomal synthesis, coupled with further posttranslational modifications has also been documented for a few oligopeptide classes [37,38,39]. Unlike ribosomal products, non-ribosomal oligopeptides are assembled by large multifunctional enzyme complexes, commonly non-ribosomal peptide synthases (NRPS) or hybrid NRPS/PKS (polyketide synthases), which are in turn encoded in large gene clusters with a modular architecture. In general, each biosynthetic step is encoded in a single module, whereas each module is comprised by several catalytic domains [40]. 

Non-ribosomal pathways are pivotal in (cyano)bacterial secondary metabolism. NRPS genes may account for up to 5% of the genome (e.g., [41,42]). Notably, the set of proteins comprising a complete NRPS synthetic pathway can be twice the size of the ribosome. Whereas the ribosome translates thousands of different proteins, NRPSs produce only small compounds. The transcription and translation of these enzyme complexes and their modular architecture imply enormous metabolic expenses for the producing cell [43,44]. Each NRPS module consists of approximately 1000 to 1500 amino acids and is responsible for the incorporation of a single monomer into the end-product’s peptide chain. Despite the burdens to maintain this massive biosynthetic machinery, non-ribosomal pathways are believed to be of ancestral origin and have been conserved and exploited in distantly related linages [45]. Therefore, the selective forces fueling their maintenance are expected to be accordingly high. In this regard, some authors have suggested that such cellular burdens are likely compensated by the modular architecture of these pathways, which allow the producing organism to profit from module reshuffling (e.g., module reorganization, duplication or skipping, recombination, point mutations, *etc*.), as a mechanism to introduce metabolic novelty with minimal genetic changes [46,47]. A representative example of such metabolic plasticity in cyanobacteria is the gene cluster encoding the synthesis of the hepatotoxin nodularin, which evolved from the microcystin gene cluster through the deletion of two modules, thereby giving rise to a brand new end-product [48].

In addition, single NPRS gene clusters can be responsible for the synthesis of multiple chemical variants (e.g., >100 microcystin variants have been described). A significant part of this structural variability is achieved by relaxation of substrate specificities, resulting in variable incorporation of amino acids at some residues and thereby leading to the existence of multiple chemical variants synthesized by the same enzyme complex [49,50]. Substrate promiscuity, together with further modifications like methylation, glycosylation, halogenation or sulfatation at different residues [34,51,52,53,54] allow for the existence of hundreds of congeners within the same peptide class, whose minor structural differences often result in differences in their bioactive properties [55,56,57]. Both the modular architecture of encoding gene clusters and the high structural diversity of their end-products indicate that non-ribosomal pathways show high intrinsic plasticity, which confers (cyano)bacteria an enormous metabolic versatility and arguably counterbalances the high cellular costs associated to their synthesis. 

Oligopeptides are widespread in at least four of the five cyanobacterial taxonomic orders or subsections [26]. However, production abilities among conspecific strains show a highly uneven distribution [58,59]. This has been attributed to frequent horizontal gene transfer, recombination and gene loss events affecting the respective gene clusters [36,54,60,61]. As a result, strains under the same taxonomic affiliation, yet presenting dissimilar oligopeptide profiles, are commonly found in natural populations. In fact, the distribution of oligopeptide production abilities do not match with phylogenies based on extensively used phylogenetic markers (e.g., 16S-23S rRNA ITS or *cpc*BA) [19,62], or the geographical origin of the strains [61,63]. Similarly, taxonomic affiliation or morphological features do not correlate with the widely variable oligopeptide cellular compositions. Instead, single chemotypes can be represented by different morphospecies and *vice versa* [19,20,64,65]. It has, thus, become evident that traditional taxonomic systems to classify cyanobacteria, despite recurrent revisions, are unable to tackle the true extent of cyanobacterial metabolic biodiversity.

## 3. Typing of Cellular Oligopeptide Patterns by MALDI-TOF MS

The rapid development of bioinformatic tools has contributed to the increased discovery of new microbial secondary metabolites in the last years (e.g., [66,67,68]). New sequencing technologies (e.g., pyrosequencing), genome mining, and metagenomics have substantially increased our ability to identify novel NRPS and PKS gene clusters in microbial genomes. Alternatively, analytical methods based on Tandem Mass Spectrometry (e.g., LC/MS-MS), which yield increasingly higher levels of resolution, are especially useful for the separation of unknown compounds from complex natural matrices and the subsequent elucidation of their chemical structures (e.g., [35,36,69]). The potential of these techniques to further contribute to the discovery and characterization of new microbial metabolites is unquestionable. However, with regard to the use of metabolite patterns as biomarkers, these techniques do not proof particularly useful for metabolite typing at the individual level, mainly due to commonly laborious sample preparations or long analysis times. 

Instead, Matrix Assisted Laser Desorption/Ionization–Time of Flight Mass Spectrometry (MALDI-TOF MS) has become the technique of choice for chemotyping applications. MALDI-TOF MS enables a rapid determination of intracellular constituents from fresh biomass. As a result, this technique has been increasingly used for the analysis of taxon-specific microbial metabolite patterns for the rapid identification of infective or pathogenic bacterial taxa [70,71]. Similarly, MALDI-TOF MS allows for the rapid analysis of oligopeptide compositions from cyanobacterial specimens for chemotaxonomic purposes [58,72,73]. 

MALDI-TOF mass spectrometry consists in the ionization, separation and detection of analytes. A small amount of fresh cell biomass (e.g., individual colonies/filaments) is mixed with a co-crystallizing matrix. Most commonly used matrices are low weight, organic, aromatic acids, usually 2,5-dihydroxy benzoic acid (DHB) or α-cyano-4-hydroxycinnamic acid (CHCA), that are dissolved in a mixture of solvents like water, ethanol and acetonitrile, and acidified by a strong acid, usually trifluoracetic acid [73]. Upon solvent evaporation, matrix crystals begin to form, embedding proteins and other cellular constituents (*i.e*., co-crystallization). A high energy beam laser is then focused on the sample and ions are thereby produced. The co-crystallizing compound partially absorbs the energy of the ionizing laser, allowing for a soft ionization that maintains the integrity of the molecules during analysis and producing easily identifiable singly-charged ions. Ions are accelerated in an electromagnetic field and guided through a drift-free area, where they travel at different velocities depending on their respective mass/charge ratios. Analyte separation is thereby achieved. Accelerated molecules eventually collide against a detector that provides a resolved mass spectrum. 

A commonly highlighted limitation of MALDI-TOF MS is that analysis only yields qualitative (or semi-quantitative) results, as peptides differ in their ionization due to differences in amino acid compositions and hydrophobicity. For example, peptides containing arginine in their structure show preferential ionization [74]. Similarly, some oligopeptide classes like microcystins and cyanopeptolins are readily ionized and thereby easily detected by MALDI-TOF MS, whereas other peptide classes, such as microviridins or cyclamides produce only poor signals and may be difficult to detect by MALDI-TOF MS. For these reasons, MALDI-TOF mass signals do not directly reflect actual cellular peptide concentrations, hampering quantitative interpretations of the mass spectra [75]. Despite this limitation, the indisputable advantage of MALDI-TOF MS compared to other competing techniques is speed of analysis. MALDI-TOF MS enables the typing of cellular oligopeptide compositions in single cyanobacterial specimens (e.g., filaments or colonies) within minutes. Hundreds of samples can be analyzed overnight using automated routines, allowing for high throughput analyses without complex sample preparation and at relatively low cost. Fresh biomass can be directly analyzed and only a small amount is needed to obtain satisfactory mass spectra. Besides, in contrast to MALDI MS, competing molecular techniques based on the amplification of genes responsible for biosynthesis are unable to provide information on the chemical diversity of the end-products.

## 4. Suitability of Oligopeptides as Biomarkers and Definition of Chemotypes

Oligopeptides are suitable biomarkers of cyanobacterial subpopulations, as their synthesis at the individual level is determined by the presence or absence of the respective gene clusters. Given the patchy distribution of these clusters among co-existing strains, cyanobacterial cells can present any combination of these clusters and thereby exhibit distinctive qualitative oligopeptide compositions, which delineate different chemotypes [58,59]. Cyanobacterial isolates have been observed to produce the same set of peptides for decades [19] and oligopeptides remain largely within the producing cells and are only significantly released upon cell lysis [76]. Furthermore, because peptide synthesis is constitutively regulated and peptides exhibit only slight fluctuations in cell quota [77,78,79], it seems evident that overall oligopeptide synthesis is genetically, rather physiologically, controlled. Therefore, it is generally assumed that oligopeptides are consistent subpopulation markers and consequently, that the pheno/chemotype faithfully represents the genotype as far as NRPS/PKS gene clusters is concerned. 

Several studies have evaluated the stability of oligopeptide patterns as reliable subpopulation markers under a range of environmental conditions. Whereas these studies report overall stability of peptide fingerprints, in some cases, nutrient limitation, high irradiance [80,81] and cyanobacterial cell densities [82] were observed to induce distortions in oligopeptide signatures of some strains of the genera *Microcystis* and *Radiocystis*. In particular, nutrient deprivation and high light resulted in the disappearance of minor microcystin variants (*i.e*., low intensity spectral signals) from the peptide fingerprints, presumably due to physiologically induced reductions in oligopeptide synthesis. Alternatively, several aeruginosin, cyanopeptolin, and microcystin variants could only be detected at high cell densities, remaining otherwise undetectable under low cell concentrations [82]. Difficulties in consistently detecting minor mass signals by MALDI-TOF MS had been reported earlier [56,83], although they were not interpreted in the context of the delimitation of chemotypes. Variations in peptide patterns are likely attributable to physiological fluctuations in overall oligopeptide synthesis at the individual level. For example, nitrogen inputs [84] and intracellular availability of free amino acids [85] were shown to impact microcystin intracellular shares by promoting the synthesis of some variants over others. Similarly, oligopeptide cellular concentrations, although constitutively regulated, exhibit up to five-fold fluctuations under changing conditions [78,86]. These variations, although minor, may turn out critical to detect minor peptides present at cellular concentrations close to analytical detection limits. In fact, peptides accounting for distortions in oligopeptide fingerprints are usually accompanied by one or more signals corresponding to major congeners of the same peptide class [80,81,82]. However, in the context of the definition of chemotypes, inconsistent detection of minor oligopeptides has to be treated with caution, especially when investigating chemotypes directly from natural populations. Under natural conditions, physiologically-induced variability in the detection of minor peptides might suggest shifts in the phenology of chemotypes under unchanged clonal compositions, thereby acting as a potential cause of confusion. 

Perhaps due to such inconsistencies, existing studies using oligopeptides as subpopulation markers often apply heterogeneous criteria for chemotype delineation, each of which offers a dissimilar trade-off between resolution and stability. Criteria based on (1) single oligopeptide classes (e.g., [87]); (2) “strict” individual-oligopeptide-based chemotyping (*i.e*., chemotypes display identical oligopeptide profiles, [20,63]); and (3) “lax” individual-oligopeptide-based chemotyping (*i.e*., chemotypes share resembling, but not necessarily identical peptide compositions, [19,65]) coexist in the literature. However, harmonized benchmarks for the definition of cyanobacterial oligopeptide chemotypes seem necessary, especially if comparisons among studies are to be made. In this regard, assessing the appropriateness of chemotype definitions based on single oligopeptide variants compared to criteria based on general structural classes seems crucial. The significance of structural ambiguities inherent to oligopeptide synthesis plays an important role in this question. In this regard, studies undertaking structure-activity relationship (SAR) analysis have repeatedly reported changes in oligopeptide bioactivity resulting from structural variability: cyanopeptolin moieties exhibit differences in protease inhibition capabilities [56], whereas methylations [55] and amino acid variability at some microcystin residues [88] result in changes in chymotrypsin and phosphatases inhibition, respectively. Similarly, aeruginosin variants differ in their inhibitory properties of different groups of serine proteases ([57] and references therein). These findings indicate that oligopeptide unique moieties, variable amino acid residues and general structural variability affect the bioactive properties of oligopeptides and determine their degree of specificity and activity. Therefore, chemotype definition based on individual oligopeptide variants likely encompasses functionally dissimilar subpopulations with regard to their bioactive potential and seems therefore preferable to chemotyping criteria considering general peptide classes. 

Chemotype delineation based on individual peptide variants, however, reveals a picture of high complexity. The extent of structural variability throughout oligopeptide classes could be enormous. Microcystins present over 100 structural variants, whereas other oligopeptides may bear even more structural variability. Cyanopeptolins, for instance, display variable amino acids at all positions, except for two highly conserved residues. One could hence argue that the potential structural diversity of oligopeptides, considering combinatorial incorporation of amino acids and modifications at variable residues across peptide classes, can give rise to up to hundreds, if not thousands of individual oligopeptides, and, thus, may lead to a virtually endless number of chemotypes. However, field studies have shown that naturally-occurring chemotypes delineate intraspecific linages that present unique ecological features [19,20] and, therefore, the distribution of oligopeptide production abilities is unlikely a random process. Instead, it arguably constitutes an adaptive response to selective pressures exerted by their environment. 

## 5. Distribution, Composition and Dynamics of Chemotypes in Natural Populations

Research addressing the composition and dynamics of cyanobacterial oligopeptide chemotypes has revealed that natural populations exhibit a mosaic structure of coexisting strains with different oligopeptide production abilities. Chemotype assemblages showed to be stable over for long periods of time in their respective ecosystem, as evidenced by the continuous presence of four different *Planktothrix* chemotypes in a Norwegian lake for over 30 years [19]. In contrast, the relative abundances of chemotypes in the population are not static and individual subpopulations are subject to strong fluctuations over the season, leading to marked temporal dynamics. The seasonal succession of chemotypes does not follow any apparent cyclic trends, although, in light of their long-term stable coexistence, periodic interseasonal patterns cannot be discarded. 

As a result of the different chemical profiles among coexisting strains, the phenology of individual chemotypes dynamically affects the properties of the whole-population with regard to average oligopeptide contents [19], including hepatotoxic peptides like microcystins. Fluctuations in toxin loads are of obvious relevance from the water management and public health perspectives. In fact, cyanobacterial blooms are well known for exhibiting variations in microcystin concentrations of up to several orders of magnitude in space and time [89,90,91]. Such differences cannot be explained by physiological changes, as toxin production at the individual level varies within a narrow range [92]. Instead, it has become evident that the wax and wane of toxigenic and non-toxigenic chemotypes is the factor driving bloom toxicity [20,65,91]. Therefore, elucidating the mechanisms governing the complex succession of chemotypes is crucial, not only to identify the factors that promote more toxic blooms, but also to interpret cyanotoxin occurrence in an ecological context. 

Tracking individual oligopeptide-based subpopulations in their natural habitat revealed that cyanobacterial chemotypes delineate subpopulations that interact differently with their environment [19,20]. The annual life-cycle of planktonic colonial cyanobacteria of the order Chroococcales, such as the bloom-forming genus *Microcystis*, is well described. Toward the end of the blooming season colonies settle down to the sediments, where they spend the winter in the so-called overwintering phase. Toward spring, benthic colonies regain buoyancy and are recruited back into the water column, giving rise to the summer pelagic population, thereby closing the cycle [93]. In this regard, recent findings indicate that individual chemotypes may go through shifts in their life-cycle with different outcomes. First, the comparison of benthic and pelagic compositions of chemotypes during the season revealed notorious differences, with some chemotypes exclusively present as benthic subpopulations [20]. Habitat segregation is indicative of dissimilar recruitment success among chemotypes. Benthic recruitment is considered a major process determining the size and composition of summer pelagic populations (e.g., [94]). Therefore, although recruitment in genus *Microcystis* is traditionally supposed to be triggered by physical factors (e.g., light, temperature, sediment resuspension, or bioturbation), chemotype segregation among benthic and pelagic habitats indicates that reinvasion might be more complex than previously described and suggests that recruitment might operate as a selective process that shapes strain compositions in benthic inocula. In fact, promoted recruitment of microcystin-producing strains has been reported in genus *Microcystis* [95,96], supporting the idea that chemotypes might as well display different success rates reinvading the water column. 

Over the course of the season, the temporal evolution of the benthic composition of chemotypes reflects with fair fidelity the major shifts occurring in the overlaying water column [65]. This indicates that chemotype dynamics are mostly driven by differential settling among subpopulations. In fact, later quantitative studies identified asynchronous sedimentation among co-existing chemotypes as the primary contribution to the succession of chemotypes in the water column [20]. Much like in the case of benthic recruitment, evidence for differential settling of co-existing subpopulations seriously challenges views considering autumnal sedimentation as an unspecific process homogenously affecting the whole-population as a result of essentially physical phenomena, such as attachment of clay particles to colonies [97] or low temperatures [98]. 

In light of these considerations, we believe that the ecological relevance of the subspecific level in cyanobacteria as operating basis for biological process is indisputable. Therefore, revisiting the ecology and life-cycles of cyanobacteria under perspectives that recognize the intraspecific dimension of their populations is needed to increase our understanding on how the interplay of ecological processes dynamically shape the populations along their life-cycle at its different stages. Moreover, addressing differential behaviors among chemotypes in their environment constitutes a promising way to identify the selective forces driving their dynamics and thereby unravel the biological role of oligopeptides in the producing organisms.

## 6. Insights into the Biological Role of Oligopeptides and the Evolution of Chemotypes

The notion that chemotypes represent ecologically relevant units raises important research questions: Which factors drive the succession of chemotypes in nature? What is the adaptive value of oligopeptides in the ecology of chemotypes? Furthermore, what forces are behind the subdivision of cyanobacterial populations into distinct chemotypes? Do chemotypes represent discrete evolutionary units? If so, what is their evolutionary history? In our opinion, these questions delineate current frontiers of knowledge on the topic. 

The adaptive value of cyanobacterial oligopeptides remains, despite intensive research, still ambiguous. A number of biological functions have been proposed, including their role as defense compounds against grazers [56,99,100,101], allelopathic metabolites against eukaryotic algae [102,103,104], info-chemicals involved in quorum sensing [105,106], or compounds involved in bloom termination [107]. However, none of these hypotheses has reached full consensus and the role of cyanobacterial oligopeptides is still a matter of intense scientific discussion. 

Traditional approaches to address this issue typically focus on individual peptides to investigate their effects on the producing organisms or their competitors. Alternatively, cyanobacterial chemotyping constitutes a complementary approach based on the study of oligopeptide-based subpopulations to infer a general biological role of these compounds. Combined interpretations of the findings stemming from these approaches have contributed to the formulation of novel hypotheses on the adaptive value of oligopeptides that are consistent with observations at different organizational levels (e.g., population and molecular levels). 

Chemotypical subpopulations in their environment can be controlled either by bottom-up or top-down mechanisms. Bottom-up mechanisms are mediated by resource competition among subpopulations. However, although a role of oligopeptides as chelating agents putatively enhancing nutrient uptake has been proposed [108,109], attempts to correlate the prevalence of chemotypes with abiotic factors like temperature, light or macronutrients turned out unfruitful [19,20,78]. This is coherent with the co-occurrence of chemotypes in geographically distant water bodies with widely different morphologies and trophic states [63,110]. Altogether, these findings cast doubts on the likelihood of bottom-up control mechanisms effectively shaping chemotype communities. 

Alternative notions for the existence of top-down mechanisms controlling cyanobacterial populations are not new. However, grazing and competition with other phytoplankton have been typically pointed out as their possible drivers. In fact, putative roles of oligopeptides as grazing deterrents [56,99,100] or allelopathic compounds against eukaryotic algae [104] have been intensively explored. However, the fact that oligopeptides remain within the producing cell and are only released upon cell lysis [76,111] has often been raised as a strong argument against this possibility; an effective allelopathic or defensive effect could only take place upon cell death or ingestion and the benefits of such “post-mortem” protection are questionable. 

Other proposed forms of top-down control refer to parasitic interactions. Parasites in aquatic ecosystems have traditionally been neglected, mostly due to methodological limitations. At present, however, host-parasite interactions are increasingly regarded as fundamental in the functioning of the ecosystem and considered important driving forces of ecological and evolutionary processes, such as species succession, population dynamics or gene flows [112,113,114]. Parasites are also relevant ecosystem components that enhance the transfer of nutrients and energy to higher trophic levels and contribute to the recycling of organic matter [115]. Parasites can impose strong top-down control on host populations. Cyanobacteria are no exception and they are indeed targeted by potent pathogens like phages [116] and parasitic fungi [117], which can inflict significant mortality on cyanobacterial populations. Among the latter, parasitic fungi of the order Chytridiales, commonly referred to as chytrids, are regarded as important parasites of cyanobacteria, whose infection is considered as an omnipresent phenomenon in aquatic ecosystems [117]. Chytrid parasites display absorptive nutrition; they encyst at the host cell surface and form intracellular rhizoids to extract nutrients from the host [118]. In the process, chytrids secrete digestive proteases into the host cell [119,120,121]. Cyanobacterial hosts, however, are endowed with an intracellular cocktail of oligopeptides, most of which display potent enzyme inhibitory properties that often target proteolytic enzymes: Oligopeptides belonging to the cyanopeptolin, aeruginosin, anabaenopeptin, and microginin classes display diverse inhibitory effects on a range of proteases (e.g., [27,122,123,124,125,126,127,128]). In fact, in a recent study, *Planktothrix* mutants unable to synthesize microcystins, anabaenopeptins and microviridins, respectively, displayed higher susceptibility to chytrid infection, compared to the wild-type strain [129]. These findings convincingly demonstrate that cyanobacterial oligopeptides are effectively involved in defense mechanisms against chytrid parasites, most likely by inhibiting chytrid proteases involved in pathogenicity. In contrast to grazing protection or allelopathy, an anti-fungal defensive role is compatible with the intracellular compartmentalization of oligopeptides. 

Interestingly, further studies exploring the relationship between cyanobacteria and their fungal parasites reveal that chytrid strains present chemotype-specific host preferences [130]. The fact that chemotypes display different susceptibility to co-existing chytrid parasites and that such susceptibility responds to different combinations of intracellular inhibitory oligopeptides, led to the formulation of evolutionary scenarios to explain the subdivision of cyanobacterial population into different chemotypes. According to evolutionary theory, host and parasite are caught in a close antagonistic relationship of reciprocal adaptations, commonly defined as an evolutionary arms race [131]. Since parasites typically have shorter generation times than their antagonists, and hence display higher evolutionary rates, hosts cannot keep pace and are strongly selected toward diversification [132,133]. By diversifying, hosts hamper the ability of parasites to optimally exploit the whole population. This theoretical framework was first proposed by Sonstebø and Rohrlack [130] to explain the subdivision of *Planktothrix* populations into distinct chemotypes. Under this scenario, individual chemotypes are considered evolutionary units that co-evolve with chytrid parasites. Narrow host range and high chemotype specificity of chytrid fungi are consistent with co-evolutionary models, whereas chemotype subdivision is also compatible with predicted host diversification as a strategy to resist rapidly evolving parasites. 

In cyanobacteria, predicted host diversification might be reflected, first, at the population level by the co-existence of chemotypical subpopulations with dissimilar susceptibility to parasites. Rather than maintaining an ideal genotype, cyanobacteria may profit from preserving an array of chemotypes to prevent parasites from exploiting the whole population efficiently. The dynamic composition of chemotypes might therefore respond to top-down pressures exerted by parasites or, alternatively, other pathogens (e.g., cyanophages). Secondly, signs of diversification are also evident at the peptide level. It is somewhat notorious that during their evolutionary history, the vast pool of peptide congeners has not been reduced to a few efficient ones. Instead, oligopeptide synthesis is characterized by widespread structural ambiguities that result in the production of a wide array of peptide variants, which in fact exhibit different inhibitory properties [55,56,57]. As discussed above, relaxation of substrate specificities during biosynthesis accounts for a significant part of such variability. In-depth analyses of the oligopeptide encoding gene clusters evidenced that the majority of the cluster is under purifying selection and therefore highly conserved. However, small genic regions presenting relaxed selective constrains could also be identified [45,61,134,135]. Interestingly, some of these sites are located close or within adenylation domains (*i.e*., domains mediating for the activation and incorporation of amino acids into the growing peptide chain), which are responsible for amino acid hypervariability during biosynthesis. This suggests that domains responsible for substrate promiscuity might be under positive selection and hence, the synthesis of new oligopeptide variants might be promoted, thereby further contributing to the diversifying potential of oligopeptide repertoires. 

Differences in oligopeptide profiles arguably imply dissimilar fitness costs among coexisting subpopulations, which likely result from energy burdens, but also from antagonistic pleiotropy associated with oligopeptide synthesis [136]. Intraspecific competitive differences among chemotypes are therefore likely to exist. However, considering the diversity of chemotypes in nature, these differences do not seem to lead to the competitive exclusion of chemotypes in the long term. Conversely, stable chemotype consortia are observable over decades in the same system [19]. Considering top-down control mechanisms mediated by parasites, the concept of “killing the winner” introduced by Thingstad and Lignell [137] provides a plausible explanation for the long-term maintenance of the diversity of chemotypes observed in natural populations. According to this idea, the superior competitor, which is expected to be the most abundant subpopulation, would be most intensively decimated by the pool of coexisting parasites. This negative feedback exerts a stabilizing effect, preventing the exclusion of otherwise less competitive subpopulations and maintaining chemotype diversity over time, in spite of competitive differences among subpopulations. This process is compatible with field observations reporting that shifts in the mosaic composition of cyanobacterial populations, which are typically characterized by the dominance of one or a few abundant chemotypes [20,65], are often reflected as the decline of the dominant chemotype, with minor chemotypes taking over. In fact, chemotype-specific losses selectively inflicting massive cell-lysis-related mortality to the dominant chemotype have recently been reported [20]. These selective loss processes are compatible with the chemotype-specific preferences of chytrid strains reported in laboratory experiments [130] and support the existence of parasitic top-down control, coupled with massive epidemics as stabilizing mechanisms behind the long-term maintenance of chemotype diversity. 

While some aspects of this novel hypothesis definitely remain to be experimentally demonstrated, we believe that the coherence between the available pieces of evidence (at both the population and molecular levels), and the theoretical framework explaining the emergence of intraspecific chemotypes is too striking to be overlooked and deserves serious consideration in future research.

## 7. Conclusions and Future Research

The use of oligopeptides as cyanobacterial markers is still in its infancy, but has strongly contributed to tackle the chemical variability among co-existing cyanobacterial strains. High throughput analytical techniques like MALDI-TOF MS allow the rapid analysis of qualitative oligopeptide compositions at the individual level and, thereby, enable the delimitation of chemotypical subpopulations. Oligopeptides proofed to be in general suitable subpopulation markers, given their constitutive synthesis and their steady intracellular concentrations. The analysis of chemotypical subpopulations under natural conditions has revealed that cyanobacterial populations are comprised by a stable mosaic of coexisting chemotypes that are subject to marked seasonal dynamics. Close examinations of the succession of subpopulations in aquatic ecosystems show that chemotypes interact differently with their environment and present different ecological traits. The existence of ecologically functional units below the species level stresses the relevance of the subspecific dimension as the basis on which biological processes operate and shape cyanobacterial populations. Current research in this direction is focused on the identification of the selective forces governing the dynamics of chemotypes in nature, to disentangle the biological role of oligopeptides and to interpret cyanotoxin occurrence in an ecological context. Recent findings stemming from chemotyping approaches strongly suggest that cyanobacterial peptides are involved in defense mechanisms against fungal parasites and, most likely, other pathogens. Cyanobacterial highly diversified oligopeptide repertoires and population subdivision into distinct chemotypes have been proposed as adaptive strategies of cyanobacteria to resist rapidly evolving parasites. Whereas this novel hypothesis on their biological role remains to be experimentally confirmed and will likely shape future research directions, the use of oligopeptides as biomarkers exemplifies how much we can learn by leaving behind traditional notions considering the species as the ecologically relevant unit.
